# Supplementary material for: Chromothripsis during telomere crisis is independent of NHEJ, and consistent with a replicative origin
Source: Genome Res. 2019 May;29(5):737–49. doi: 10.1101/gr.240705.118 (PMC6499312; doi:10.1101/gr.240705.118)
Supplement: Supplemental Material [file supp_gr.240705.118_Supplemental_file_1.zip › contigs/annotated_contigs/DB55/contig.2.DB55_length_431_mean_cov_5.12993039443.docx]

**DB55_length_431_mean_cov_5.12993039443**

```
CAGCGGACTCGGGCGCGCCTTTGTCTGAGCTGCGGTGCGCCGGGAGTCCTGCGCCTTTGTGTGACCCTCGCAGGTGTGCGAGCGGAGGT
 >chr19:42637366-42637582 - E=2e-118
GTTGGGAGGGTGTTGGGGAGGGGAGCGGAGGGAGGGGCTCCCTTCTCCGCAGCCGGCCCCGCCCTCCGGCCCATTGGCTGGGCGCTGCC

GCCCTCTGGCGGGCACGGAGGGAAGCGCTGAAATG|GGA|CCTGTGCAGCGTGTGCATAATAGACACAGAACTACCTACGACTCCCTGT
                                    >chr19:42683951-42684169 + E=1e-116
ACGGAGCAGCCTCCTGGGACCCGCACTGTGTGCGCAACACAAATAAATGCAAGGAATACTACCCTCTGTCCATACAAAGTAGGTATTTG

GGTCTTATGCAGCAGATGCATAATGGACATGGGACCACTTAATACCCCCATACAGAGCAGACTCCTGGGATCCACAC
```
